# Societal impact of dengue outbreaks: Stakeholder perceptions and related implications. A qualitative study in Brazil, 2015

**DOI:** 10.1371/journal.pntd.0005366

**Published:** 2017-03-09

**Authors:** Joël Ladner, Mariana Rodrigues, Ben Davis, Marie-Hélène Besson, Etienne Audureau, Joseph Saba

**Affiliations:** 1 Rouen University Hospital, Epidemiology and Health Promotion Department, Rouen, France; 2 Axios International, Paris, France; 3 Paris Est University, Hôpital Henri Mondor Hospital, Public Health, Assistance Publique Hôpitaux de Paris, Créteil, France; Common Heritage Foundation, NIGERIA

## Abstract

**Background:**

The growing burden of dengue in many countries worldwide and the difficulty of preventing outbreaks have increased the urgency to identify alternative public health management strategies and effective approaches to control and prevent dengue outbreaks. The objectives of this study were to understand the impact of dengue outbreak on different stakeholders in Brazil, to explore their perceptions of approaches used by governmental authorities to control and prevent dengue outbreaks and to define the challenges and implications of preventing future outbreaks.

**Methods:**

In 2015, a qualitative study was conducted in two urban states in Brazil: São Paulo, which was experiencing an outbreak in 2015, and Rio de Janeiro, which experienced outbreaks in 2011 and 2012. Face-to-face interviews using a semi-structured questionnaire were conducted with nine different categories of stakeholders: health workers (physicians, nurses), hospital administrators, municipal government representatives, community members and leaders, school administrators, business leaders and vector control managers. Interviews were focused on the following areas: impact of the dengue outbreak, perceptions of control measures implemented by governmental authorities during outbreaks and challenges in preventing future dengue outbreaks.

**Results:**

A total of 40 stakeholders were included in the study. Health workers and community members reported longer waiting times at hospitals due to the increased number of patients receiving care for dengue-related symptoms. Health workers and hospital administrators reported that there were no major interruptions in access to care. Overall financial impact of dengue outbreaks on households was greatest for low-income families. Despite prevention and control campaigns implemented between outbreak periods, various stakeholders reported that dengue prevention and control efforts performed by municipal authorities remained insufficient, suggesting that efforts should be reinforced and better coordinated by governmental authorities, particularly during outbreak periods.

**Conclusion:**

The study shows that a dengue outbreak has a multisectorial impact in the medical, societal, economic and political sectors. The study provides useful insights and knowledge in different stakeholder populations that could guide local authorities and government officials in planning, designing and initiating public health programs. Research focused on a better understanding of how communities and political authorities respond to dengue outbreaks is a necessary component for designing and implementing plans to decrease the incidence and impact of dengue outbreaks in Brazil.

## Author summary

Since the beginning of the 21st century, dengue fever has been a significant vector-borne arboviral disease; actually more than 3.9 billion people are at risk of infection in 128 countries. Dengue has become an increasing public health concern in Latin America, especially in Brazil, which has the highest incidence rate of dengue. Researches are needed to gain in-depth understanding of stakeholder and community reactions to outbreak and to explore the societal impact of dengue outbreaks. In 2015, a qualitative study was conducted in two urban states in Brazil, which experienced recent outbreaks. Longer waiting times at hospitals due to the increased number of patients receiving care for dengue-related symptoms were reported, but without interruptions in access to care. Various stakeholders reported that dengue prevention and control efforts performed by municipal authorities remained insufficient. The consequences of a dengue outbreak reach far beyond the patients, undermining medical, social, economic and political sectors. Research focused on a better understanding of how communities and political authorities respond to dengue outbreaks is a necessity for designing and implementing plans to control dengue outbreaks.

## Introduction

Since the beginning of the 21^st^ century, dengue fever has been a significant vector-borne arboviral disease, occurring mainly in tropical and sub-tropical countries where more than 3.9 billion people are at risk of infection in 128 countries [1–4]. With an estimated 400 million annual dengue incidence worldwide, the disease is currently endemic in more than 125 countries in Africa, the Americas, South East Asia and other regions in the world [2]. Sixty percent of dengue cases occur in the Americas, predominantly Latin America, where the disease has re-emerged owing to re-infestation by the dengue vector. Since its re-emergence in Latin America, dengue has spread dramatically throughout the region [5]. The number of reported dengue cases rose from 1,033,417 in the 1980s to 2,725,405 in the 1990s, and 4,759,007 between 2000 and 2007 [2, 5]. Between 2001 and 2009, six countries accounted for more than 75 percent of all cases in the region: Venezuela, Brazil, Costa Rica, Colombia, Honduras and Mexico [6, 7]. Various political, environmental and social factors influenced the re-emergence of the disease in Latin America. Dengue is largely an urban disease [7]. Overcrowding, uncovered water sources and climate change are optimal conditions for geographic spread of the mosquito vector for dengue, which lives in domestic settings and can breed in very little water and survive drought conditions [1]. The impact of urbanization is particularly significant in low and middle-income countries [8–10].

Among the South American countries of Argentina, Brazil, Chile, Paraguay and Uruguay, Brazil has the highest incidence rate of dengue with 294 cases per 100,000 inhabitants in 2014 [11]. Brazil is considered a tropical country in its entirety because of its hot and humid climate, which provides a favorable environment for proliferation of the dengue vector. Incidence of dengue in Brazil has frequently been high, and the number of cases in the country has sometimes represented as much as 60% of all reported dengue cases worldwide [11]. After the disruption of its vector control program, Brazil experienced a series of dengue outbreaks [5, 11] characterized by increasing geographic spread and severity as estimated by the number of hospitalizations and deaths [11]. In the first eight months of 2015, Brazil experienced an increase in dengue of 140 percent over the same period during the previous year (1,416,179 cases from January to August 2015 versus 589,107 cases in 2014). During that same period, the number of severe cases increased dramatically (1,284 severe cases in 2015 versus 664 severe cases in 2014) as did deaths (693 deaths in 2015 versus 407 deaths in 2014) [11].

Dengue imposes significant economic and societal burdens on countries where the disease is endemic and, as such, estimating the associated disease impact can help inform policymakers and assist them in setting priorities for disease control and management strategies [12, 13]. The effects of dengue on health and preventive care, its economic burden and social impact on populations have not been clearly studied. Understanding dengue burden from societal and socio-economic perspectives is crucial for allocation of limited scarce public health resources among competing health threats, as well as ensuring cost-effectiveness of integrated dengue prevention and control methods.

One such method promoted by the World Health Organization to overcome challenges associated with conventional single-intervention approaches is Integrated Vector Management (IVM). Defined as a “rational decision-making process for the optimal use of resources for vector control,” IVM considers five key elements in the implementation of IVM to prevent vector-borne diseases such as dengue: advocacy, social mobilization and regulatory control; collaboration within the health sector and with other sectors; integration of non-chemical and chemical vector control methods; evidence-based decision-making; and development of adequate human resources, training and career structures [14]. The growing burden of dengue and the challenges of preventing dengue outbreaks (including increase in prevention costs such as on vector control) have increased the urgency of the public health sector to identify alternative management strategies [15–19]. Monitoring of public impact, perceptions and behaviors is needed in order to guide the development of adequate and effective strategies to controlling and preventing dengue outbreaks. Establishing key performance indicators is important in measuring the effectiveness of existing and current surveillance and vector programs. In addition, identifying and understanding societal, cultural and environmental factors related to dengue outbreaks may provide insight for the development of targeted dengue prevention and control interventions, including community involvement and engagement in vector control activities given that community-based interventions have been found to impact vector indices [20]. Furthermore, understanding dengue vector-virus-disease behaviour and human-animal-environment interactions and interdependencies, known as “One Health,” is critical to IVM [21]. This can provide early and timely added value for consolidated and harmonized mitigation and adaptation tactics and advocacy in local settings [22].

Given the dearth of literature, qualitative studies are needed to gain in-depth understanding of stakeholder and community reactions to a dengue outbreak and its related impact. The growing burden of dengue in Brazil presents an interesting model for qualitative research around the challenges and issues that need to be addressed to strengthen dengue prevention and control interventions in Brazil and throughout Latin America.

The objectives of this study were to understand the impact of dengue outbreak on different stakeholders in Brazil, to explore stakeholder perceptions of approaches used by governmental authorities to control and prevent dengue outbreaks and to define the challenges and implications of preventing future outbreaks.

## Methods

### Study area

This qualitative study was conducted from April to August 2015. The study sites for collecting responses to interviews with stakeholders were the municipalities of Marília and Sorocaba in São Paulo State and the municipalities of Búzios and Rio de Janeiro in Rio de Janeiro State. Both states are densely populated, urban areas located in southeast Brazil, characteristics favorable for dengue outbreaks. Both of these states experienced recent epidemic dengue outbreaks, defined as at least 300 cases per 100,000 inhabitants [19]. The 2015 outbreak in São Paulo State was concurrent with the study and had an incidence of 3,762 and 9,072 inhabitants per 100,000 in the municipalities of Marília and Sorocaba, respectively. Rio de Janeiro State experienced a series of outbreaks that had surmounted by the time of the study. Rio de Janeiro experienced outbreaks with incidences of 1,090.5 per 100,000 inhabitants in 2011 and 567.7 per 100,000 inhabitants in 2012 [11]. Búzios experienced outbreaks in 2007 and 2011 with incidences of 2,007 and 1,994 inhabitants per 100,000, respectively, for each year [11].

### Selection of participants

A convenience sampling method was used to select target participants at the two research sites. With the goal to obtain responses from a diverse set of representative stakeholders affected by dengue outbreaks, various stakeholders were defined and recruited. A sample of 30–50 stakeholders across the two sites was determined to be sufficient to qualitatively address the study objectives. Stakeholder categories were selected based on the type of individuals expected to best address questions related to the study objectives. Nine categories of stakeholders were identified: municipal government representatives, hospital administrators, physicians, nurses, community members and leaders, school administrators, business leaders and vector control managers. The categories of hospital administrators and health workers (physicians and nurses) were subdivided into two sectors, public and private. Stakeholders were identified from communities in each study site particularly affected by a dengue outbreak based on incidence data reported in local Ministry of Health dengue bulletins and community recommendations. Specific stakeholders in public and private hospitals within those communities were targeted based on their level of involvement in the dengue outbreak response. Community members and leaders, school administrators and business leaders were targeted to capture a diversity of opinion, particularly as it pertains to economic background.

### Data collection

A semi-structured questionnaire was used for interviews, which focused on a range of topics regarding the impact of dengue outbreaks and response to the outbreak. Data were collected through face-to-face interviews conducted by three Brazilian social researchers. These social researchers were trained by Axios International and were based in each study site.

Interviews focused on impact of dengue outbreaks and stakeholder opinion of dengue outbreak prevention and control efforts implemented by governmental authorities. The impact of dengue outbreaks was assessed in the following areas: healthcare infrastructure and case management, municipal government operations and finances, household operations and finances, and communities (including schools and businesses). The duration of the interviews ranged from 30 to 40 minutes. All the interviews were audio recorded, then transcribed and translated from Portuguese to English. Interviews were conducted in stakeholder settings and at places that were convenient for the participants, such as their home or workplace. The data on costs of dengue prevention were obtained from Brazilian Government websites [23, 24].

### Analysis

A directed content analysis approach was used to analyze the data and to identify the key themes. Audio transcriptions and interview notes were reviewed and summarized by two members of the study team. They reviewed all qualitative comments extracted from the questionnaires and placed them into broader categories based on content and theme. All data were systematically analyzed. Key findings and quotes were compiled in a Microsoft Excel table and coded according to topic or theme. Responses were reported if at least two persons gave a similar response. Final decisions on comment categories were discussed with a third author. The research team identified quotes that best illustrated common themes and included these quotes in the results of this study. A certified translator translated quotes into English. Because the results of interviews were quite similar across both states (and the four municipalities), the decision was made to pool the results.

### Ethics statement

Participation in this study was voluntary and all participants provided written informed consent prior to the interviews. All information was collected anonymously and the outcomes were used only for research purposes. Data of the interviews are housed in Axios International, Paris, France. The Western Institutional Review Board (WIRB) approved the research (#1-904491-1).

## Results

### Participants

A total of 40 stakeholders were included in the study, with 18 stakeholders in São Paulo State (Marília and Sorocaba municipalities) and 22 in the Rio de Janeiro State (Búzios and Rio de Janeiro municipalities). The different stakeholders included in the study were physicians (Phy), nurses (Nu), hospital administrators (HA), community members (CM), community leaders (CL), school administrators (SA), municipal policy makers (MPM), vector control managers (VCM) and business leaders (BL) (Table 1).

**Table 1:** Categories of stakeholders included in the study, April—August 2015, Brazil.

Type of participants	Municipalities (number)	Total number
Community members	Sorocaba (8)	11
	Rio de Janeiro (3)	
Community leaders	Sorocaba (4)	7
	Buzios (1)	
	Rio de Janeiro (2)	
Physicians	Buzios (1)	4
	Rio de Janeiro (3)	
Nurses	Sorocaba (1)	4
	Buzios (2)	
	Rio de Janeiro (1)	
School administrators	Sorocaba (1)	3
	Buzios (1)	
	Rio de Janeiro (1)	
Hospital administrators	Sorocaba (1)	3
	Buzios (1)	
	Rio de Janeiro (1)	
Municipal government leaders	Marilla (1)	3
	Rio de Janeiro (1)	
	Buzios (1)	
Vector control manager	Buzios (1)	2
	Rio de Janeiro (1)	
Business leaders	Sorocaba (2)	3
	Rio de Janeiro (1)	

The study included three physicians and three nurses from a public facility and one physician and one nurse from a private facility. Two hospital administrators were from a private facility and one from a public facility. One nurse and one physician from separate private hospitals also had previous experience working in a public emergency care center during a dengue outbreak.

### Impact of dengue outbreaks

#### On healthcare infrastructure and case management

Between January (2,561 confirmed cases) and February 2015 (13,563 confirmed cases), Sorocaba dealt with a 430 percent increase in confirmed dengue cases within a month. In March, at the peak of the outbreak, the city reported more than 22,000 confirmed cases. As a result, the rapid influx of patients reporting dengue symptoms quickly overwhelmed both public and private healthcare facilities in the city. “*It was scary*. *Both public and private service centres were overflowing*. *The urgent care centres in private hospitals were also over capacity*.” (HA1).

Healthcare facilities faced difficulties in finding the necessary space to care for all incoming patients during the outbreak, often moving patients between facilities or referring them to centres further from their homes believed to be better equipped to handle the demand. In addition, staffs on call were not always sufficient to meet patient demand, leading to stress, fatigue and unexpected absenteeism.

Case notification and follow-up processes also became increasingly difficult to follow in outbreak environment. *“To notify a certain pathology*, *you need to have time*. *Many times*, *the number of patients that you see is so extensive that you do not have the time to fill out a mandatory notification form for each patient*. *There is a huge demand and I think that it’s because of it that the number of dengue cases are not always notified properly”* (Phy2).

Anxiety and fear of contracting dengue further contributed to demand for care services. “*During the months with the highest number of dengue cases*, *patients become extremely anxious and afraid of receiving a dengue diagnosis*. *Almost all of them asked for complete blood work examinations or some other specific type of examinations*, *even if it was not recommended by the doctor*. *The huge demand of patients asking for these types of tests really slows down the intake process for all patients who are waiting to be seen*. *So it ends up affecting everything*. *It’s like a domino effect”* (Phy4).

The increase in the number of patients attended in the hospital during a dengue outbreak had a direct impact on availability of beds and waiting time. The rapid patient influx was reported to have resulted in delayed diagnoses for dengue and other diseases, but was not reported to have an impact on care and treatment provision. “*Dengue treatment in itself is not complicated*. *A problem is only evident during an outbreak*, *when the volume of cases spikes”* (Phy1). *“Dengue outbreak does have an impact on the diagnosis of other diseases due to increased patient load in emergency rooms*. *The impact is increased waiting times for all patients*. *However*, *apart from increase waiting time*, *there is no impact on the actual treatment of other diseases during a dengue outbreak”* (Nu3). The number of patients during an outbreak was also seen to contribute to the overall cost of managing the disease at the health facility level. *“It's not an expensive management on a patient-by-patient basis*. *Dengue ends up becoming expensive because it is a disease that has a large number of cases*.*”* (Phy4).

Community members also reported longer waiting times at hospitals during an outbreak. “*During an outbreak*, *there can be longer waiting times to be seen at the health posts due to the number of people seeking treatment*” (CM6). “*There is an increase in hospital waiting times during a dengue outbreak*” (CM4). “*The waiting time was so long in the clinic closest to my house that I had to take my wife to several clinics before I found one where the waiting time wasn’t so long*” (CM5).

Physicians and nurses reported a strong increase in informal complaints from patients, most of which were related to procedural changes and increase in waiting times. “*The hospital did receive more formal complaints from patients during an outbreak period*, *usually due to changes in procedures which are necessary to handle the increased patient load*. *Most were informal complaints*. *There were few formal complaints directed to the municipality*.” (Phy4).

From the point of view of hospital administrators, dengue outbreaks did not appear to have a strong impact on daily hospital management and on the care of patients not being treated for dengue-related symptoms. “*During a dengue outbreak*, *there is no important impact for the treatment of other diseases in the hospital*” (HA1). Hospital administrators reported that dengue outbreaks did not cause shortages of health supplies (including blood supplies) and a process of reallocation of different health resources specifically for dengue was not required. “*The most recent dengue outbreak did not interrupt healthcare services or the treatment of other diseases*. *It did not lead to a shortage of resources and it did not lead to a reallocation of resources*” (HA2).

Healthcare professionals with experience working in both the private and public sector reported facing more significant patient management challenges in the public sector. “*When I worked in the emergency care unit*, *we faced staff shortages*. *All the employees were overworked and the doctors barely had time to eat*, *leading to waiting times that were much too high*. *This was tiring for the staff and the patients*” (Nu2). “*Patients in the public sector face enormous challenges*, *including the lack of hiring of temporary staff*. *There is no shortage of staff*, *the hospitals are just not able to hire because the public facilities don’t receive additional funding and this contributes to a chain of events that lead to the outbreak*” (Phy1). The referral of dengue cases to public facilities, which were described as more experienced with the management of dengue patients, particularly severe cases, was another reason provided to explain differences between public and private facilities during an outbreak.

#### On households

Community leaders indicated that a dengue outbreak had a direct impact on households, particularly if the infected member of the family was the female head of the household (as opposed to the male head). “*The impact when a female head of household is diagnosed with dengue is greater than when a male head of household is diagnosed with dengue because she is usually responsible for running the household*” (CL5).

Overall financial impact of dengue outbreaks on households was limited, but greatest for low-income families. “*The impact of dengue in terms of direct costs is scanty (even cheap compared to other healthcare costs)”* (CL3). *“A dengue outbreak is not very inconvenient or costly”* (CM8). *“I do not view the missed days of work/school as a major cost”* (CM1). “*Even though the cost of dengue care is minimal*, *it had an impact on low-income populations*” (CL1). Community members reported purchasing a range of mosquito control methods during an outbreak, such as citronella candles, insect swatters, special plants, sand to fill empty receptacles and insect repellents. Insect repellents were the most often cited method of prevention among higher-income respondents, while community members of lower-income populations did not frequently mention it. In regard to treatment, dengue care and treatment was largely free in healthcare facilities. At the beginning of a dengue outbreak, self-medication rates were high. Patients would transition from self-medication to clinics as symptoms worsened. During the course of treatment for a dengue infection, costs for transportation were estimated to be US$2 to $4 per person. Transportation costs were reported as the most significant expenditure for low-income households.

#### On schools

School administrators indicated higher levels of absenteeism during dengue outbreaks. Increased absenteeism was reported among both students and teachers. During outbreaks, absenteeism ranged from 10 to 15 percent for both teachers and students. Approaches used by schools to confront absenteeism during dengue outbreaks included sending educational materials home (either by physical delivery or email) and having teachers prepare materials and lessons in advance in case of student absence. “*The school has numerous strategies to enable students who are sick for extended periods not to fall behind”* (SA2). School administrators noted that schools did not receive additional funding for dengue prevention and control measures.

Schools also adapted their curriculums to address community sensitization needs during a dengue outbreak. Educational materials and lessons on dengue prevention and control were introduced into the curriculum, with a specific focus on identification and removal of mosquito breeding sites and personal care. Meetings with parents were held to educate them about dengue prevention, and to reassure parents that schools were taking proper control measures. “*Education is a key tool to prevention in the long term*” (SA3). For schools in areas with high illiteracy rates, social mobilization in the classroom provided an additional means to reach parents through students.

#### On businesses

Business leaders reported that the impact of dengue outbreak on local businesses was restricted. They noted that there were some increased costs for businesses since some companies paid the costs of personal mosquito control (such as repellents) for their employees. *“In the recent dengue outbreak*, *there has been little impact on the day-to-day function and sustainability of the business*” (BL1). “*Some companies paid for mosquito repellents for their employees”* (BL2).

#### On municipal governments

In response to the growing demand for care and treatment during an outbreak and the resulting community dissatisfaction with government dengue prevention and control efforts, municipal governments intensified their efforts. Additional physicians and other healthcare staff were appointed, temporary medical units were established and necessary medical equipment was purchased, rented or borrowed from other facilities. Municipal budgets were specifically allocated for the reinforcement of prevention and control of dengue. State of emergencies declared during dengue outbreaks provided additional powers to municipal governments to make decisions regarding vector control activities and policies.

As the incidence of dengue escalated, growing fear and anxiety for contracting the disease significantly impacted political structures. Community dissatisfaction and mistrust for government prevention and control efforts increased and fuelled a high level of community disengagement with these efforts. Media also played an influential role in community reaction to the outbreak. Articles in international media focused on the magnitude of the outbreak and its impact on growing political tensions, while national and local media outlets concentrated on the direct impact on Brazilian communities and were largely triggered by the release of new epidemiological bulletins or government announcements. Leaders of municipal governments perceived clearly the potential of serious political impact during dengue outbreaks and believed that outbreaks could lead to serious political breakdown. “*A dengue outbreak can lead to a political breakdown*, *despite the best efforts of leaders”* (MPM1).

Inadequate community support for government prevention and control efforts, combined with a reported lack of accountability by the community, may have limited the cost-effectiveness of these efforts. In Sorocaba municipality, a total of R$ 7.5 million (US$ 2.6 million) was spent on dengue control between January and May 2015 (the end of the outbreak was announced on May 20), with an estimated R$ 11 million (US$ 3.5 million) to be spent in total by the end of 2015 (22, 23).

### Stakeholder opinions of control efforts implemented by the authorities during outbreaks and challenges in preventing future outbreaks

#### Community leaders and members

Community members generally reported negative opinions of methods used by municipal governments to control dengue outbreaks. They felt that the preventative and control measures were either not taking place, not sufficient or were done too quickly to be effective. They described the system as not being ready to handle health emergencies, dengue or otherwise. Community leaders also thought that municipal governments were slow to react and to implement interventions during dengue outbreaks. They believed that there was insufficient preparation for quick and efficient intervention resulting in unnecessary delays. “*In the beginning*, *the local government thought that the situation was not going to last long and then things got worse*. *In other words*, *they did not work on preventing the issue and once the outbreak started*, *they were not prepared to deal with it*. *The government took the necessary actions only when things had already escalated*” (CL3). Sorocaba residents cited house visits, fumigation trucks and public education as the most visible control and prevention measures by the government. However, feedback was inconclusive in relation to the frequency of these activities and varied significantly by neighbourhood and period of outbreaks. “*The greatest impact is the lack of confidence of the population towards the public authorities where healthcare is concerned*. *This one is still causing ripples*” (CL2).

While community members, leaders and healthcare practitioners interviewed agreed that controlling dengue was a shared responsibility of the government and the community, most felt that residents did not take the necessary responsibility over the issue. “*You know that here in Sorocaba*, *just like all over Brazil*, *the population is at fault in dengue fever propagation*. *Because they leave containers without lids full of water out in open air*. *They leave waste in their backyards*. *They are concerned about the outbreak*, *but they do not try to solve the problem” (CL4)*. However, some community leaders felt that the community did a reasonable job of uniting for the common good during dengue outbreaks. “*While there is an absence of more government intervention*, *the community does a reasonable job of uniting for the control of dengue outbreak*” (CL1). “*We needed more agents focused on prevention*, *so we took it upon ourselves*. *I am part of a group that provides education to the community on dengue*. *As community leaders*, *we stepped in to help*” (CL5).

Community leaders emphasized that there needs to be more support for dengue outbreak prevention programs from the government. “*There needs to be more support in dengue outbreak prevention from the government*, *especially in regard to more health agents who go door-to-door to educate and check for mosquito breeding sites* (CL2)*”*.

#### Health workers

Physicians and nurses pointed out that official preventative actions were inadequate and remained critical. *“I think that the dengue prevention and control efforts provided by the government are still insufficient” (N3)*. “*The city is taking preventive measures*, *but these measures are not yet adequate to prevent and control dengue*” (Phy1).

#### Hospital administrators

Hospital administrators thought that dengue prevention and control efforts performed by the government and municipal authorities were insufficient, suggesting that prevention efforts needed to be better coordinated and reinforced, specifically between the outbreak periods. “*I think they (city government) managed to resolve the existing demand*. *Regarding prevention however*, *I believe there’s still a gap*” (HA1).

#### Municipal governments

Interviews with municipal government leaders defined awareness building and community mobilization as the greatest challenges to overcome during dengue outbreaks. *“Prevention efforts cannot be successful without the mobilization of the community”* (MPM1). Municipal government leaders said that education played a major role in reducing outbreaks. “*The greatest challenge during the outbreak was increasing awareness of the population and mobilizing them”* (MPM3). “*The major need to prevent a dengue outbreak is an increase in education”* (MPM2).

#### Vectors control managers

Vector control managers, responsible for identification and eradication of mosquito breeding sites, stated that community resistance was an issue and that additional community education around dengue prevention and control could help prevent dengue outbreaks and improve the effectiveness of vector control efforts like fumigation. *“People always ask my team when they see us why we don’t come more often with our fumigation trucks*. *But these are the same people that close their windows when our trucks pass*, *making our job practically obsolete”* (VCM1).

## Discussion

The study shows that a dengue outbreak undermines medical, social, societal, economic and political stability through a rapid patient influx into the healthcare system, the financial burden on governments and households, and the effect of community dissatisfaction and disengagement on outbreak control measures. This multi-sectorial impact, although limited, results in social and political disruption. In addition to providing a description of the challenges that need to be surmounted during dengue outbreaks, this study suggests important considerations that should be accounted for in the design and implementation of future dengue prevention and control interventions.

For the majority of stakeholders interviewed in this study, dengue control and prevention efforts implemented by the authorities were seen as insufficient. As citizens witnessed the rapid growth of dengue cases in their communities, fear and anxiety for contracting the disease impacted social structures. Stakeholders expressed frustration over the lack of concerted and timely government efforts to prevent and control outbreaks in the community, and many did not perceive the impact of such interventions. Stakeholders also felt that prevention efforts did not appear to be sufficiently coordinated. Insufficient governmental response or late responses to outbreak management appeared to generate a lack of confidence from the community. As a result, communities became increasingly disengaged and uninterested in vector control efforts in their household or neighbourhoods. Our findings underline the importance of permanent and strong social mobilization efforts and concerted community interventions in dengue prevention and control and the difficulty in maintaining mobilization efforts in the community over time [19].

In regards to healthcare system, the significant and rapid influx of patients placed a major burden on public and private health facilities as they struggled to find the space and the staff to handle demand and the means to minimize increasingly long patient waiting times. Planning around potential outbreak scenarios prior to an outbreak and improving dengue case management in health facilities during outbreak periods is a priority in ensuring improved clinical outcomes and quality of care. This is particularly critical in cities with limited infrastructure and human resources.

The study provides a significant description of the challenges that need to be surmounted during future dengue outbreaks and suggests important multifocal approaches that should be developed to meet these challenges. Indeed, the results of this qualitative study mirror studies done in other regions in the world, such as Southeast Asia, Ecuador and Mexico, with regard to economic burden and effects on community and municipal functions [13, 25, 26]. Poor populations are greatly impacted and approaches to education need to be improved. A better community understanding of approaches to dengue control is important to their successful implementation [8, 14, 27, 28]. However, development of these educational programs requires knowledge of the culture, organization and functioning of community members and local authorities [14, 28, 29]. Such knowledge can be obtained by exploring the community’s level of understanding of dengue outbreaks obtained from information communicated by governmental authorities, media (newspapers, radio, TV) and social networks.

To better manage and increase confidence of community members in vector control actions, protocols specifically designed for the local environment that can be rapidly implemented at the beginning of an outbreak need to be further developed [30]. Planning should include improved approaches to educating community members and leaders about dengue, increasing the availability of dengue resources before outbreaks and maintaining necessary resources and services between outbreaks. During outbreaks, local sensitization campaigns must focus on community mobilization activities to eradicate mosquito breeding. Knowledge or awareness has been reported as being important for the success of dengue prevention and control efforts, and inadequate knowledge about dengue was found to be a major impediment to the successful implementation of approaches used to eliminate dengue. Importantly, studies indicate that dengue knowledge alone did not single-handedly translate to adoption of preventive measures. Instead, policy makers and planners must also focus sensitization campaigns on reducing barriers to behaviour change related to control of dengue fever among the population [16, 31], and encouraging communities to adopt preventive measures [16]. Such actions should increase community confidence in government responses to dengue outbreaks while decreasing fear and misconceptions. Increasing the confidence of community members would also be expected to decrease the chances of political breakdown, a concern of community and government leaders during an outbreak.

Dengue prevention and control should include individuals, families and the wider community and encourage community participation to improve its chances for success. In a routine dengue prevention and control program, a community based environmental strategy showed a significant reduction in levels of *Aedes* infestation by 50–75% compared with a routine program without community involvement [19]. Comprehensive and enhanced dengue intervention strategies based on community engagement could have a significant and effective impact on dengue outbreak control, yet are highly dependent on the community’s perception of the severity of the disease. Intervention strategies should be built into existing health care systems and closely coordinated with the national dengue control program [29, 30].

The cost of dengue control at the household level also needs to be addressed in relevant protocols and community mobilization activities, particularly with regard to the allocation of resources during an outbreak and with planning for poorer populations. In this study, lower income populations also reported more challenges in covering the cost of treatment, or difficulty paying indirect costs, such as transportation charges for hospital or health facility visits [13].

While this study was not intended to quantify the economic impact of the disease, the financial data gathered highlights the substantial investment needed to cover the cost of on-going prevention and control efforts. At least R$ 4.2 billion (US$ 1.28 billion) was spent on dengue control and prevention efforts between 2010 and 2014 in Brazil [23, 24]. At the federal, state and municipal levels alike, the financial burden of dengue is significant and multi-sectoral. State and federal contributions may not always be sufficient to meet municipal prevention and control needs. For example, Sorocaba’s spending in 2015 for dengue control and prevention was an estimated R$ 11 million (US$ 3.5 million) for the full year, nearly twice the total amount distributed by the state to all 645 municipalities in São Paulo [32, 33]. The results also emphasize the critical challenge around financial accountability for dengue control and prevention among levels of government and the limited impact of costly year-round campaigns. LR Carasco *et al*. demonstrated that the average economic impact of dengue illness in Singapore from 2000 to 2009 ranged between US$0.85 billion and US$1.15 billion, of which control costs constitute 42%–59% [34]. A 2013 study conducted in 12 countries in Southeast Asia showed that, there was an annual average of 2.9 million (m) dengue episodes and 5,906 deaths from 2001–2010. The annual economic burden (with 95% certainty levels) was US$950 million (US$ 610million—US$ 1,384million) or about US$1.65 (US$ 1.06 —US$ 2.41) per capita. A study conducted in four Brazilian regions estimated that the cost for dengue prevention and control for the epidemic season in 2012–2013 was substantial [35]. The annual national economic burden was US$ 164 million from the public payer perspective, but may be as high as US$ 447 million (adjusted for underreporting). From the societal perspective, the economic burden was US$ 468 million, but may be as high as US$ 1,212 million [35].

In addition to the medical, economic, social and political impact of dengue, this study also found that another key factor contributing to the need to prioritize dengue is that it is a highly visible disease. This visibility is due to the fact that dengue often occurs in epidemics, which attracts media attention, stokes public fear and puts a strain on municipal budgets. This is in contrast to non-epidemic diseases, such as diarrheal disease and pneumonia, which attract less public or media attention [36]. For this reason, community engagement and satisfaction is particularly important in the dengue context. Furthermore, given the visibility of the dengue, the role of the media during dengue outbreaks should be better understood. Appropriate and effective management approaches, ready to deploy before a dengue outbreak occurs, would decrease the burden on health care facilities, increase the confidence of community members in government responses to outbreaks and would keep municipalities operating with increased efficiency. Increased confidence of community members would also be expected to improve community involvement and compliance with official approaches to dengue control. Improving community involvement and compliance during dengue outbreaks should decrease the intensity and duration of the outbreak and decrease the chances of subsequent outbreaks [37].

Recently, a long-term follow-up and integrated efficacy analysis of 35,000 children between the ages of 2 and 16 years in Asian Pacific and Latin American countries showed a reduction in dengue disease in the efficacy surveillance phase among children and adolescents who received a recombinant live attenuated tetravalent dengue vaccine. The vaccine was also associated with a lower risk of hospitalization and severe dengue overall up to 2 years after completion of the three-dose vaccination schedule among children 9 to 16 years of age [38]. Disease impact modelling has further indicated that if 20% of the populations in the 10 countries that participated in these efficacy studies were vaccinated, this could potentially reduce dengue burden by 50% in these countries [39]. Improved approaches to dengue management that include the considerations discussed in this study will continue to play a key role in a comprehensive prevention and control strategy. Future research should include gaining an understanding of how a vaccine would help the local community, including analysis of the cost- effectiveness of a vaccine program and the impact of vaccination as part of an integrated prevention strategy for dengue in endemic countries [35, 40]. Initiation of a public dengue immunization program could serve as a long-term and visible commitment by leaders to protecting populations against the economic and human impact of this disease, and hence may contribute to regaining public trust in governments where public confidence has been affected by a dengue outbreak.

This study presents limitations. First, the results should be considered in the context of the study sample size and the nine categories of stakeholders included, with an unequal distribution of stakeholders per category. The unequal distribution is partly associated with difficulties faced in identifying voluntary participants in select stakeholder categories directly involved in prevention and control measures given the sensitive nature of the topic at the time of the study. In certain stakeholder categories there may exist some limitations to data transferability, yet in most categories, data saturation was achieved. Secondly, since the study is qualitative and explorative, caution must be used with interpretation of the results. Further investigations will require other methodological approaches such as quantitative methods to confirm our findings. In addition, while the value of focus group discussions in presenting varying opinions and perspectives is recognized, the sample size of the study did not permit for such an approach. Thirdly, the sample of informants may not have included all major stakeholders in each municipality or have been representative of all stakeholders. As with all qualitative studies with open-ended responses, there is also the possibility of misunderstanding or biased interpretation of participant responses. Nevertheless, the strength of this qualitative study lies in the sample comprised of a large panel of stakeholders from a diverse socio-demographic background, which closely resemble the population usually involved in dengue control. The structure of the interviews, which allowed for probing and clarification of responses, and analysis were designed to minimize misinterpretation.

In conclusion, the study provides useful insights from different stakeholder populations that could guide local authorities and government officials in planning, designing and implementing integrated public health programs and activities aimed at preventing and controlling dengue in Brazil. The consequences of a dengue outbreak reach far beyond the people infected, undermining medical, social, economic and political stability. Ultimately, an effective means of preventing dengue outbreaks that combines vector control and vaccination would reduce the social and political disruption and economic impact of the disease. The recent explosive outbreak of the Zika virus in South America reinforces the urgent need for more integrated research and public health strategies to control arboviral diseases [41, 42]. Similar to the qualitative study reported in this paper, research that is designed to understand how a community and its local government responds to dengue outbreaks and two other arboviruses (chikungunya, Zika) circulating in Brazil, is a necessary component for designing and implementing plans to decrease the incidence of dengue outbreaks in Brazil. Such research will inform public policy by providing evidence-based recommendations to decrease the burdens of dengue outbreaks on both communities and governments

## Supporting information

S1 Checklist STROBE checklist. (DOC)
